# Value of peer mentoring for early career professional, research, and personal development: a case study of implementation scientists

**DOI:** 10.1017/cts.2021.776

**Published:** 2021-04-08

**Authors:** Kelsey S. Dickson, Joseph E. Glass, Miya L. Barnett, Andrea K. Graham, Byron J. Powell, Nicole A. Stadnick

**Affiliations:** 1 Department of Child and Family Development, San Diego State University, San Diego, CA, USA; 2 Child and Adolescent Services Research Center, San Diego, CA, USA; 3 Kaiser Permanente Washington Health Research Institute, Seattle, WA, USA; 4 Department of Counseling, Clinical & School Psychology, University of California, Santa Barbara, CA, USA; 5 Department of Medical Social Sciences, Northwestern University Feinberg School of Medicine, Chicago, IL, USA; 6 Brown School and School of Medicine, Washington University, St. Louis, MO, USA; 7 Department of Psychiatry, University of California, San Diego, La Jolla, CA, USA; 8 UC San Diego Altman Clinical and Translational Research Institute Dissemination and Implementation Science Center, La Jolla, CA, USA

**Keywords:** Peer networking, implementation science, case study, career development, early-stage investigators

## Abstract

Effective mentoring is a key mechanism propelling successful research and academic careers, particularly for early career scholars. Most mentoring programs focus on models pairing senior and early career researchers, with limited focus on peer mentoring. Peer mentoring may be especially advantageous within emerging areas such as implementation science (IS) where challenges to traditional mentoring may be more prevalent. This special communication highlights the value of peer mentoring by describing a case study of an early career IS peer mentoring group. We delineate our curriculum and structure; support and processes; and products and outcomes. We highlight important group member characteristics to consider during group formation and continuation. The group’s long-term (6 years) success was attributed to the balance of similarities and differences among group members. Members were in a similar career phase and used similar methodologies but studied different health topics at different institutions. Group members gave and received instrumental and psychosocial support and shared resources and knowledge. Peer mentoring can serve an important function to provide emotional, logistical, and professional development support for early career scholars. Our case study highlights strategies to foster peer mentoring groups that provide a generalizable blueprint and opportunity for improved outcomes for early career professionals.

## Introduction

Receipt of mentoring, particularly early in one’s career, can promote successful transition to professional independence [1]. It facilitates networking within one’s field, which is critical to establishing and expanding one’s research program [2]. Mentorship also has immense value for populations who face unique career challenges, including women and marginalized individuals [3,4]. Thus, mentoring is key to the successful professional development of early career researchers. The objective of this special communication is to describe a case study of a peer mentoring group of eary career implementation scientists to highlight the value of peer mentoring and examples of how to structure a peer mentor network. In the following sections, we provide an overview of mentoring, the rationale for focused attention on peer mentoring, and our group’s spectific goals, processes, and outcomes.

### Prioritizing Mentorship for Early Career Implementation Scientists

Mentoring is a critical ingredient for conducting innovative, impactful translational research, including implementation science (IS). The National Institutes of Health (NIH) and National Science Foundation (NSF) recognize the importance of mentorship in facilitating professional success [5]. The National Institute of Mental Health (NIMH) National Advisory Council stated, “Effective mentoring, which is often lacking, is one of the elements essential to the development of a successful research career” [6]. The importance of mentoring for early-stage implementation scientists is underscored by increasing numbers of competitive national and international IS training programs prioritizing mentored training (e.g., Implementation Research Institute; Mentored Training in Dissemination and Implementation Research in Cancer; Training Institute in Dissemination and Implementation Research in Health of the USA, Ireland, and Australia) [7–10]. Similarly, mentorship is a core feature of US grant mechanisms for early-stage investigators, such as NIH Career Development Awards (i.e., K awards), NSF Faculty Early Career Development Program, and the Institute of Educational Sciences Early Career Development and Mentoring programs.

### What Gaps Exist in Typical Mentoring in Research Settings?

Traditionally, mentoring is a unidirectional, hierarchical relationship between an established, senior mentor providing mentoring to a less established, junior mentee, most commonly co-located in the same institution [11]. In this traditional pairing, mentors may benefit from the mentoring process [12], but the benefits are primarily realized by the mentee. There are career benefits to obtaining diversified mentoring, such as receiving guidance from multiple mentors, bidirectional mentor–mentee relationships within and outside of one’s field and institution and from individuals at various career levels [7,9,11]. There are also benefits unique to a variety of mentoring delivery formats (e.g., formal and informal, individual and group-based). Cross-institutional mentorship enables more equitable and comprehensive access to the benefits of mentorship, as some seeking mentors may be disadvantaged due to having limited mentor availability or expertise at their institution. Several IS training programs facilitate the acquisition of cross-institutional mentorship in multiple formats, including peer mentoring [2,7,10,13]. Yet, there is little guidance on how to conduct IS peer mentoring, especially outside of the context of formal training programs.

### What Is Peer Mentoring and Is It Effective?

**Fig. 1.**
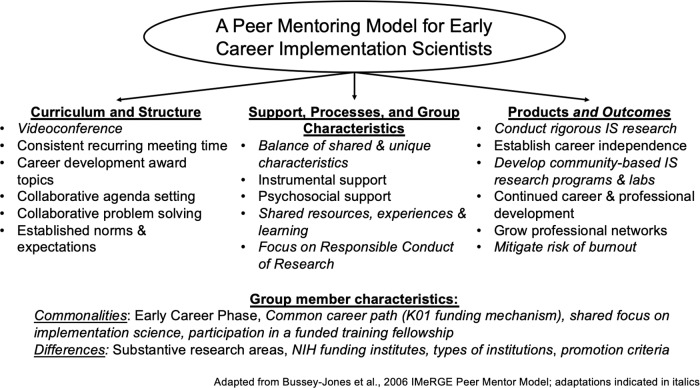
Guiding peer networking model. IS, implementation science; NIH, National Institutes of Health.

Peer mentoring, or bidirectional mentoring between those at a similar career stage, is a well-established professional development practice, especially during career stages that are characterized by development and transition [14,15]. An extant model of peer mentoring highlights key components of peer mentoring: having a core curriculum, opportunities to give and receive support, and the promotion of activities leading to products and outcomes (see Fig. 1) [16]. Peer mentoring should increase access to and use of other mentoring structures and promote professional development [15]. It may also offer unique psychosocial benefits, including friendship and emotional support from colleagues with shared experience and/or career stage [15]. Peer mentoring has the potential to complement traditional hierarchical mentorship models such as overcoming issues related to retention of early career academics, and it can address capacity limitations of senior mentors [17].

### Why Peer Mentoring Is Particularly Useful for IS

Peer mentoring is especially important for implementation scientists who face both typical early career challenges and challenges specific to conducting IS research. Despite rapid growth, IS remains a relatively new field, resulting in limited availability of collaborators and senior mentors [18]. Peer mentoring addresses this challenge by shifting the task of mentoring from senior mentors to the growing number of junior investigators. Further, the collaborative and cross-disciplinary nature of IS as well as unique methodology calls for additional training from those with distinct backgrounds, expertise, and institutional affiliations [18], and peer mentoring is well suited to supporting expertise development in these areas. Indeed, IS trainees cite peer mentoring as a highly valued way of developing these research skills and networks [2,9]. Given its potential to optimize training, facilitate collaborations, and accelerate research, peer mentoring holds promise as a tool in promoting early and sustained success of future implementation scientists.

### Purpose

We present a case study of a peer mentoring group featuring early career implementation scientists to highlight the value of peer mentoring for those in positions that require the production of rigorous scholarship and the acquisition of extramural funding. The case study is based on our experience in an ongoing peer mentoring group. Each of us hold K awards, which support early-stage investigators in establishing their scientific independence through mentored training and research. Though hierarchical mentoring is a requirement of these awards, peer mentoring is not, and we formed this peer group to supplement mentorship from senior colleagues. To develop this case study, we engaged in a group discussion and consensus processes to identify core components that fostered success of the group. Our case study is informed by the IMeRGE peer mentoring model [16] and literature on mentorship in clinical and translational science [19]. Specifically, the IMeRGE model specifies recommended peer mentoring methods (e.g., member responsibilities, curriculum, member support) that facilitate early career collaboration and success [16]. We describe the origin and growth of this group, structure and format, core components, and qualitative impact on professional, personal, and academic domains. Insights from this case study could inform the development of peer mentoring groups aiming to foster and support the career development of early career implementation scientists. While we detail the creation, process, and perceived impact of our peer mentoring group, our intent is not to be prescriptive but to share our experiences as a potentially useful model of peer mentorship.

## IS Peer Mentoring Case Study

### Group Origin and Expansion

This IS peer mentorship group emerged organically after two members, M.B and N.S., started biweekly one-on-one conversations as postdoctoral fellows to address challenges and progress related to applying for NIH K awards in 2015. The desire to share common professional and scientific struggles among peers was further revealed upon meeting other fellows of NIH-funded training networks, including the Child Intervention and Prevention Services Fellowship (http://chipsfellows.com/) and Implementation Research Institute (http://iristl.org/about/) [7]. Networking opportunities within these formal training programs facilitated connections with two more NIH K awardees, effectively transforming the modality to a small group format in 2017. Over time (2018–2019), two additional K awardees joined the group. Each of the six members of our group are from a different US institution (two from schools of medicine, one from a school of social work, two from a school/college of education, one from a research institute embedded within a healthcare delivery system). Each member joined as assistant professors/investigators during the first year of their K award, and all focus on behavioral health and IS.

### Curriculum and Structure of Peer Mentoring Activities

Consistency, routine, and having established expectations have been foundational to our group’s curriculum and structure (Fig. 1 adapted from the IMeRGE model). We meet monthly for 1 hour via videoconference. Group discussions balance encouraging flexibility in topics while maintaining a general structure and guiding principles for each meeting. Meetings start with collaborative agenda setting to identify and prioritize topics that meet the developmental needs of the group. Common agenda items include developing and executing studies, concrete logistics, professional development, mentoring trainees, “managing up” with senior mentors, and psychosocial support during times of stress (see Table 1 for peer mentoring topics). Our group upholds two guiding principles. The first is use of collaborative problem-solving. Our collective experiences are used to validate one another’s experience of a problem and provide feedback in an action-oriented manner. The second is establishing norms and expectations that unequivocally center on mutual respect and promoting one another’s programs of research and career advancement.

**Table 1.** Curriculum topics from early career implementation scientist peer mentoring group

*Developing and executing studies*
Developing and maintaining community partnerships
Managing changes in research plans
Selecting appropriate implementation science research designs
Engaging with funders about study implementation
*Concrete logistics*
Completing National Institutes of Health (NIH) progress reports
Adjusting NIH clinical trials recruitment targets and clinical trial reporting
Preparing manuscripts and determining authorship decisions
*Professional development*
Leading a research lab
Applying for research funding and building a research program
Managing and growing collaborative relationships
Mentoring trainees and research staff
Maximizing relationships with senior mentors
Balancing research and faculty duties
Attending to promotion considerations and timelines
Selecting and managing professional development opportunities
Serving on grant review committees
Interviewing for and negotiating jobs and academic advancements
*Psychosocial support*
Managing the uncertainty of research conduct during COVID-19 and in response to shifts in funding organizations’ priorities
Managing disappointment and achievement related to academic products (e.g., grants, publications)
Managing changes in academic institutions
Navigating role transitions (e.g., parenthood) that impact career development

Note: The amount of time devoted to any topic in a specific peer mentoring group session shifts relative to the needs and priorities of the group members.

### Support and Processes: Keys to Success

We conceptualize supports and processes from the IMeRGE Model as keys to our success as a peer mentoring group, which also contributed to our individual professional development and may translate to the success of future groups. The first key to success is having members with a balance of shared and unique characteristics. We are in a shared career stage with common career paths (e.g., K awards) and a shared focus on IS, which was essential in fostering the support and impact of this group. We also previously participated in group-based NIH-supported training fellowships, which enabled foundational knowledge of NIH funding mechanisms and team science. However, we are located at different institutions with varying job expectations (e.g., soft vs. hard money faculty lines, teaching requirements, promotion criteria) and are funded by different NIH institutes. This enables representations of a variety of perspectives, while maintaining a shared focus on issues facing K awardees. Having members outside of one’s institution and/or outside the context of the hierarchy within one’s academic or research institution also enables discussion of key topics or issues without the potential for negatively impacting one’s professional development or evaluation. Additionally, our research programs share commonalities, with notable distinctions. Each is evaluating interventions to address a behavioral health problem, with a focus on understanding how the intervention can be delivered at scale leveraging IS methodology. This similarity permits meaningful and informed contributions to each other’s research challenges. At the same time, we target different health conditions (e.g., autism, substance use) and/or use varying intervention delivery modalities (e.g., via lay health workers, mobile devices), which encourages collaboration without inciting competition.

The second key to success is the instrumental and psychosocial support afforded by and unique to the peer mentorship structure. Because of our shared factors described above, we take advantage of the hands-on experiences we are simultaneously obtaining with our K awards and offer instrumental support in carrying out our projects and conducting responsible research. Because we are navigating this process concurrently, we often provide advice that reflects *current* NIH practices and policies for awardees relative to prior policies or practices that might be more familiar to senior mentors. Moreover, when seemingly inconsequential, mundane, or “obvious” questions arise (e.g., how to change NIH recruitment milestones, convening a Data Safety and Monitoring Board), it can be more appealing to ask these questions among peers than senior mentors or program officers. Examples include sharing information about rules related to awards, progress report completion, clinical trials reporting, and aspects of new grant applications. Sharing this knowledge allows us to strategically prepare for conversations with our program officials and mentors. Similarly, because our K awards and other funding streams are through different NIH Institutes, we disseminate learnings from discussions with our different institutes and expand our research funding networks. This group facilitates the responsible conduct of research, a focus within NIH K awards, by sharing resources and knowledge as well as feedback surrounding ethical issues that arise and often particularly unique in community-engaged IS research (e.g., equitable compensation of community partners) [20].

The peer mentorship model allows for opportunities to reflect on how to be a successful mentee to senior mentors, and mentor to junior trainees, unique skillsets that are necessary for early career researchers [21]. Relying on peer mentorship for tangible advice and support allows us to maximize our time with senior mentors to focus on substantive scientific questions and longer-term professional development and provides space to discuss topics pertinent at the early career stage that may be difficult or that cannot be shared with mentors (e.g., negotiating departmental politics, considering faculty positions at other institutions). It also allows us to solicit advice and support on how to “manage up” in our relationships with senior colleagues or mentors, that is, how to proactively receive the support needed from the mentoring relationship. There is also a substantial transfer of information across peers of what we learn from senior mentors. Because we all have unique mentoring teams, we benefit from learning strategies that have been offered by others’ mentors and allowing each of us to harness the diversity of ideas across multiple senior mentors without increasing burden on any one senior mentor. This maximizes the reach of our senior mentors’ influence, benefitting the IS field as a whole. Beyond focusing on how to be a successful mentee, peer mentorship supports development of our own capacity as mentors and scientific leaders. We often discuss how to best mentor our own trainees, including how to make individualized development plans with them, manage co-authorship, maximize research support with limited funding, develop academic projects from our research studies, build their own academic trajectories and grow diverse, equitable, and engaged teams.

Similar to how we benefit from learning from each other’s mentors, we have benefitted each other’s mentees by sharing materials (e.g., past grant awards) and enhancing networking opportunities. Overall, peer mentoring provides a form of unique psychosocial support as compared to traditional mentoring relationships. There is a sense of psychological safety afforded by peer mentoring, which allows us to ask questions that might be difficult, inappropriate, or detrimental to discuss elsewhere.

### Products and Outcomes

Based on our collective experiences, we offer examples of how peer mentoring activities have benefitted the authors, their institutions, and funders. We consulted and collaborated with one another around the development and submission of multiple published manuscripts and grant proposals that have been submitted or funded (e.g., Barnett et al. 2020 [22], Dickson et al., 2020 [23], Graham et al., 2020 [24]). We also supported other professional development activities, including serving as guest lecturers or invited speakers at each other’s institutions, nominating one another for professional awards and leadership roles in professional societies, and presenting together at conferences. Group members have also offered intellectual and administrative insights into one another’s projects without an expectation of a formal role (e.g., as a co-investigator or co-author). For example, in January 2020, the NIH released a notice indicating a new administrative supplement for K awardees to support retention during critical life events. When the notice was released, one of us had just returned from maternity leave and another announced impending maternity leave. These members consulted with each other and the group about their applications and shared feedback from our respective program officers and mentors regarding considerations for their submissions. Both were awarded the supplements. Additionally, we offer advice about approaches for disseminating learnings from our awards, such as publishing study protocols, research data, and papers that support our training goals.

In addition to direct benefits to the authors, we believe peer mentoring has benefitted our institutions and funders. We share resources such as writing guidance and examples of successful grant applications to colleagues writing K applications. Such efforts help to foster the next generation of NIH-supported early career investigators. Finally, our group has helped manage professional development issues commonly faced by early career scientists that can impact productivity and success, including changes in institution and the impact of family commitments. To this end, an important product of our group has been fostering the health and wellbeing of group members to facilitate professional independence, successes, and retention in academic careers.

## Discussion

This case study highlights the value of peer mentoring in fostering professional development and progress toward career independence for our group of early career implementation scientists. Our model (Fig. 1) emphasizes the benefits of shared and unique group member characteristics; instrumental and psychosocial support provided; and shared resources, experiences, and learning. These components contributed to numerous products and goals among its members, including continued career and professional development and successful growth of our research portfolio. These domains closely align with key areas of mentor competencies [19]. These characteristics and resulting products were greatly facilitated by the group curriculum and structure, namely the collaborative agenda setting and problem-solving, established expectations, and norms of the group. Together, the components of our peer mentoring model have enabled its 6-year sustainment and impact across multiple levels, including individuals; other identified mentors and home institutions; and collaborators and research networks.

The value of peer mentoring we have experienced is consistent with and adds to the small body of peer mentoring-focused literature [2,9] and informs peer mentoring groups aiming to support the career development of early career implementation scientists. Additionally, there is particular value for maximizing other received mentorship, including formal mentoring via K awards and other training programs. This multi-institutional form of peer mentoring could be a way to expand efforts to reduce disparities related to professional development, particularly given the historical accumulation of privilege, funding, and expertise within the top academic and research institutions [25], and the disproportionate impact of early career challenges on marginalized individuals [3,4,16]. Finally, while peer mentoring may be appropriate for all early career researchers, this form of mentoring should not serve as a substitute for more traditional hierarchical mentoring or sponsorship, as those relationships remain critical to successful career advancement.

There are a variety of potential avenues for increasing the use of peer mentorship to support early career researchers. At the individual level, we encourage researchers to proactively seek and initiate peer mentorship relationships. This could take place locally within one’s institution, but it is increasingly feasible to identify and engage peers regionally, nationally, and internationally through widely available networking tools. For example, the NIH’s RePORTER (https://reporter.nih.gov/) and associated Matchmaker tool could be used to find investigators conducting work relevant to one’s interests. Social media platforms such as Twitter or ResearchGate could also be used to catalyze peer mentorship networks. Universities and research institutions could support peer mentorship through formal structures embedded within Clinical and Translational Science Institutes, cross-institution centers and interest groups such as the Washington University Network for Dissemination and Implementation Research (https://publichealth.wustl.edu/dandi/wundir/) or University of California San Diego Altman Clinical and Translational Research Institute Dissemination and Implementation Science Center. (https://disc.ucsd.edu), and interdisciplinary fellowships such as the University of North Carolina at Chapel Hill Thorp Faculty Engaged Scholars Program (https://ccps.unc.edu/fes/). Although some perceived benefits of our model may not hold if translated to other peer mentoring groups, such as early career investigators at the same institution with similar institutional funding but differing research fields, alternative shared commonalities may yield other benefits to its members. Research funders could encourage peer mentorship by connecting researchers within their funding portfolios that are working in similar or adjacent areas, and affording opportunities for interaction through grantee meetings, research conferences, or other sponsored gatherings. Finally, professional societies may be uniquely positioned to connect early career researchers who have similar interests and goals. For example, the Society for Implementation Research Collaboration (https://societyforimplementationresearchcollaboration.org) has a mentorship program that links emerging scholars with more senior implementation scientists; recently, the student group has chosen to focus on peer mentorship to expand opportunities for mentorship to *all* students, developing students’ mentoring skills, and reducing burden on more senior researchers. However, we contend there is a need to be mindful of necessary adjustments to the process or membership of these groups to maintain the key components of peer mentoring, including engendering an equal power distribution and psychosocial safety or support.

## Acknowledgments, limitations, and next steps

It is important to acknowledge the limitations of this case study. First, our members represent a small cross section of early career researchers. Each member already had multiple advantages and career achievements prior to beginning monthly meetings. For example, by the nature of having secured NIH K awards and having participated in other NIH-funded fellowships, we had access to training and senior mentorship regarding IS, career development, and federal funding. These types of peer mentoring networks, while clearly advantageous, afforded privilege and have the potential to exacerbate disparities in regard to academic advancement, tenure and promotion, and federal funding that are seen for women and scholars of color. Given that the majority of K awardees do not secure subsequent large-scale funding (e.g., NIH R01 [26]), and disparities in federal funding for women and scholars of color persist [27,28], it could be beneficial for scholars who systemically face challenges to career achievement to have peer mentoring explicitly provided. Further, others have highlighted the importance of friendship and co-mentorship for women and other individuals who are marginalized within the academy to promote resiliency, address self-doubt, and help navigate professional challenges [29,30]. While peer mentoring may be beneficial at fostering resiliency and sharing institutional knowledge, structural changes will be needed to achieve equity. An example of the combined benefit of peer mentoring and systemic changes can be seen in our case study, where the NIH made a structural change by offering administrative supplements for those with K awards during critical life events, which could help retain early career investigators with caregiving responsibilities. Therefore, the benefits reported can likely extend to others and help address issues and/or barriers contributing to ongoing disparities for such groups. Going forward, we encourage institutions and funders to promote peer mentoring initiatives in support of early career scholar development.
